# 4-Methylumbelliferone administration enhances radiosensitivity of human fibrosarcoma by intercellular communication

**DOI:** 10.1038/s41598-021-87850-3

**Published:** 2021-04-15

**Authors:** Ryo Saga, Yusuke Matsuya, Rei Takahashi, Kazuki Hasegawa, Hiroyuki Date, Yoichiro Hosokawa

**Affiliations:** 1 Department of Radiation Science, Graduate School of Health Sciences, Hirosaki University, 66-1 Hon-cho, Hirosaki, Aomori 036-8564 Japan; 2 Nuclear Science and Engineering Center, Research Group for Radiation Transport Analysis, Japan Atomic Energy Agency, 2-4 Shirakata, Tokai, Ibaraki 319-1195 Japan; 3 Faculty of Health Sciences, Hokkaido University, Kita-12 Nishi-5, Kita-ku, Sapporo, Hokkaido 060-0812 Japan

**Keywords:** Radiotherapy, Statistical physics

## Abstract

Hyaluronan synthesis inhibitor 4-methylumbelliferone (4-MU) is a candidate of radiosensitizers which enables both anti-tumour and anti-metastasis effects in X-ray therapy. The curative effects under such 4-MU administration have been investigated in vitro; however, the radiosensitizing mechanisms remain unclear. Here, we investigated the radiosensitizing effects under 4-MU treatment from cell experiments and model estimations. We generated experimental surviving fractions of human fibrosarcoma cells (HT1080) after 4-MU treatment combined with X-ray irradiation. Meanwhilst, we also modelled the pharmacological effects of 4-MU treatment and theoretically analyzed the synergetic effects between 4-MU treatment and X-ray irradiation. The results show that the enhancement of cell killing by 4-MU treatment is the greatest in the intermediate dose range of around 4 Gy, which can be reproduced by considering intercellular communication (so called non-targeted effects) through the model analysis. As supposed to be the involvement of intercellular communication in radiosensitization, the oxidative stress level associated with reactive oxygen species (ROS), which leads to DNA damage induction, is significantly higher by the combination of 4-MU treatment and irradiation than only by X-ray irradiation, and the radiosensitization by 4-MU can be suppressed by the ROS inhibitors. These findings suggest that the synergetic effects between 4-MU treatment and irradiation are predominantly attributed to intercellular communication and provide more efficient tumour control than conventional X-ray therapy.

## Introduction

Fibrosarcoma is categorized as a rare cancer and is known as refractory malignant tumour in radiotherapy^1^. Because the tumour metastasis leads to poor prognosis, there is less evidence regarding its therapeutic effect^2^. Chemoradiotherapy has been clinically conducted to improve the therapeutic effects of fibrosarcoma. Representative chemoradiotherapy is conducted by the use of cisplatin^3^ or 5-fluorouracil^4^ to enhance the lethal effects on tumour. Other drugs for chemoradiotherapy (such as paclitaxel, gemcitabine and monoclonal antibody) have been proposed so as to efficiently eradicate tumours by using characteristics of cell synchronization, reoxygenation, and suppression of molecular targets (e.g., epidermal growth factor receptor)^5–8^. However, to realize high tumour control probability with suppressed metastasis and minimized side effects, the development of drug for chemoradiotherapy is still ongoing worldwide.

In the recent decades, it has been found that hyaluronan synthesis inhibitor 4-methylumbelliferone (4-MU) is a candidate for chemoradiotherapy involving anti-tumour and anti-invasion effects^9^. The lethal dosage of 4-MU of cancer cells (HT1080) is lower than that of normal lung fibroblast cell (WI-38), suggesting few side effects on normal tissues^9,10^.The tumour radiosensitivity under 4-MU treatment can be enhanced by suppressing inflammatory effects^11,12^. Such inflammatory responses after irradiation can be activated by intercellular signaling pathways with interleukins (e.g., IL-1β and IL-6)^13–17^, inducing both cell death in non-irradiated cells (so called “radiation-induced bystander effects” or “non-targeted effects, NTEs”)^18,19^ and radioresistance in irradiated cells (so called “rescue effects” or “protective effects”)^20–22^. In these scenarios, intercellular communication by reactive oxygen species (ROS)^23–25^ might play an important role in raising radiosensitivity of tumour in the presence of 4-MU; however the radiosensitizing mechanisms remain to be fully clarified.

To make clear the radiosensitizing mechanisms under 4-MU treatment, in vitro experiments using cancer cell line are necessary. Currently, experimental data are insufficient, and there are also limitations to clarify the mechanisms only from the cell experiments. In these circumstances, we thought that a theoretical model prediction of cell-killing in combination with the cell experiments can be a powerful approach to interpret the biological data^26–31^. Specifically, we are interested in modelling the pharmacological effects by 4-MU treatment and investigating quantitatively the radiosensitizing mechanisms by the use of an integrated cell-killing model considering several biological responses (i.e., DNA damage repair kinetics and intercellular communication)^32,33^. This approach must be of great importance as translational study between radiation biology and pre-clinical evaluation in the field of radiotherapy^31,34^.

In this study, we performed the cell experiments (clonogenic survival assay and ROS detection assay) and the model analysis for predicting tumour cell survival. Through this hybrid investigation, we show the radiosensitizing mechanisms of HT1080 cells under 4-MU treatment, which makes it possible to provide an estimation tool for predicting curative effects (cell-killing effects) after irradiation combined with 4-MU treatment in chemoradiotherapy.

## Materials and methods

### Reagents

4-MU (Nakalai Tesque, Kyoto, Japan) was diluted in dimethylsulfoxide (DMSO) (Wako Pure Chemical Industries, Ltd., Osaka, Japan) and used at concentrations of 20, 80, 100, 200 and 500 μM, and the final concentration of DMSO was 0.002, 0.008, 0.01, 0.02 and 0.05%, respectively. DMSO was also used as ROS inhibitor, and the concentration was 1%. Carboxy-PTIO (c-PTIO, Dojindo Laboratories, Kumamoto, Japan) was used as NOS inhibitor, and the working concentration was 40 μM.

### Cell culture

The human fibrosarcoma cell line HT1080 was purchased from American Type Culture Collection (Manassas, VA, USA). The HT1080 cells were grown in Roswell Park Memorial Institute 1640 medium (Thermo Fisher Scientific, Inc., Waltham, MA, USA) supplemented with 10% fetal bovine serum and 1% penicillin/streptomycin. The HT1080 cells were maintained at 37℃ in a humidified atmosphere of 5% CO_2_.

### Irradiation setup

The cultured cells were exposed to the X-rays with 150 kVp through a 0.5 mm Al and a 0.3 mm Cu filters using the X-ray generator (MBR-1520R-3, Hitachi Medical Co. Ltd., Tokyo, Japan). The dose rate measured using an ionizing chamber (Hitachi Medical Co. Ltd., Tokyo, Japan) was 1.0 Gy/min. The dose-averaged linear energy transfer (LET_D_) was estimated to be 1.53 keV/μm using a Monte Carlo simulation code, Particle and Heavy Ion Transport code System (PHITS) version 3.21^35^. In addition, the dose-mean lineal energy (*y*_D_) was estimated to be 4.68 ± 0.05 keV/μm in our previous report^36^.

### Clonogenic survival assay

The surviving fraction of HT1080 was obtained by means of colony formation assay as previously reported^9^. After seeding an appropriate number of cells on the T25 culture flasks (Thermo Fisher Scientific Inc., Tokyo, Japan), the cells were allowed to adhere 6 h prior to 4-MU administration and/or X-ray irradiation. All treatments were performed at room temperature. 10–14 days after incubated, the cells were fixed with methanol (Wako) and stained with Giemsa staining solution (Wako). The number of colonies composed of more than 50 cells was counted. The surviving fraction for each condition was calculated from the ratio of the plating efficiency for irradiated cells to that for non-irradiated cells.

### Flow cytometric analysis for detecting oxidative stress level

The oxidative stress (by ROS) level, which is intrinsically related with DNA damage induction, was measured by using DCFDA (H2DCFDA, Cellular ROS Assay Kit, abcam, Tokyo, Japan). The cells cultured in subconfluence were irradiated with X-rays and/or administrated by 4-MU at final concentration of 100 μM. The mean fluorescence intensities of DCFDA per cell were measured at 0, 2, and 24 h after treatments using a BD FACSAria Cell Sorter (BD Biosciences, Ltd., Tokyo, Japan).

### Statistics

The significance of differences between two samples was evaluated by one-way analysis of variance and the Tukey–Kramer test. The level of statistically significant difference was set to be *p* < 0.05.

### Overview of theoretical model for predicting cell survival

The present model for predicting cell surviving fraction after 4-MU treatment and/or X-ray irradiation consists of three parts: (i) pharmacological effects, (ii) DNA-targeted effects by radiation, and (iii) intercellular communication activated by radiation. The part of (i) is newly introduced in this study, whilst those of (ii) and (iii) are based on the previous modelling in *integrated microdosimetric-kinetic* (*IMK*) *model*^32^. Note that the IMK model used in this study considers dose-rate effects (i.e., cell recovery during irradiation) and intercellular communication (i.e., NTEs), which has been well verified by comparison of the model with experimental data previously reported^32,34,36,37^. We describe the details of the IMK model used in the present study in the following subsections.

### Pharmacological effects for predicting 4-MU toxicity

We first developed the IMK model for pharmacological part to estimate the surviving fraction in the presence of 4-MU. In this modelling, based on a well-known formula of pharmacological log-logistic model, the surviving fraction under drug administration, *S*_P_, is given as

1 $$S_{{\text{P}}} = S_{{{\text{Pmin}}}} + \frac{{S_{{{\text{Pmax}}}} + S_{{{\text{Pmin}}}} }}{{1 + (D_{{\text{P}}} /ED_{50} )^{rP} }}.$$

where *S*_Pmin_ is the minimal surviving fraction after drug treatment, *S*_Pmax_ is the maximum surviving fraction after drug treatment (i.e., *S*_Pmax_ = 1), *D*_P_ is the pharmaceutical dosage of drug (i.e., 4-MU concentration in μM), *ED*_50_ is effective dosage reducing cell survival to 50%, and *r*_P_ is the hillslope. It should be noted that we assumed that the suppression of cell growth by 4-MU is independent of cell-killing induced by radiation.

Using Eq. (1), we estimated cell survival curve as a function of drug dosage. The set of model parameters (*S*_Pmin_, *ED*_50_, *r*_P_) can be obtained by fitting Eq. (1) to the experimental relationship between drug dosage and surviving fraction.

### Cell-killing model considering DNA-targeted effects and intercellular communication

Second, we modified the IMK model considering DNA-targeted effects (DNA-TEs) and intercellular communication (NTEs) so as to reproduce the experimental radiosensitivities under 4-MU treatment and irradiation.

The IMK model for TEs is based on the linear-quadratic relation as function of absorbed dose of radiation; however, this model explicitly considers the microdosimetric processes and DNA damage repair kinetics during irradiation. The surviving fraction for TEs, *S*_T_, can be expressed by

2 $$\begin{aligned} - \ln S_{T} & = \left( {\alpha_{0} + \gamma \beta_{0} } \right)\dot{D}T + \frac{{2\beta_{0} }}{{(a + c)^{2} T^{2} }}\left[ {(a + c)T + e^{ - (a + c)T} - 1} \right]\left( {\dot{D}T} \right)^{2} \\ & = \left( {\alpha_{0} + \gamma \beta_{0} } \right)D + F\beta_{0} D^{2} \\ \end{aligned}$$

where *α*_0_ and *β*_0_ are the proportionality factors of the *D* $( = \dot{D}T)$ in Gy^-1^ and the *D*^2^ in Gy^-2^, respectively, $\dot{D}$ is the absorbed dose rate in Gy/h, *T* is the irradiation time in h, (*a* + *c*) represents a constant rate of sublethal damage repair (SLDR) in h^−1^^38^, *F* is the Lea-Catcheside time factor^39^ given as

3 $$F = \frac{2}{{(a + c)^{2} T^{2} }}\left[ {(a + c)T + e^{ - (a + c)T} - 1} \right].$$

*γ* is the microdosimetric quantity in Gy which includes dose-mean lineal energy *y*_D_ in keV/μm defined in ICRU report 36^40^. It should be noted that the diameter of the target packaged in cell nucleus (so called “domain”) was set to be 1.0 μm in this study. We used the *y*_*D*_ value of 150 kVp X-rays with 1 mm Al filtration (*y*_*D*_ = 4.68 ± 0.05 keV/μm) reported previously^36^. The details of the IMK model for DNA-TEs was summarized in the previous report^32^.

Next, the IMK model for NTEs considers cell death induced by intercellular signalling from radiation-hit cells to non-hit cells. The cell surviving fraction for NTEs, *S*_NT_, can be given by

4 $$- \ln S_{{{\text{NT}}}} = \delta \left[ {1 - e^{{ - \left( {\alpha_{{\text{b}}} + \gamma \beta_{{\text{b}}} } \right)D - \beta_{{\text{b}}} D^{2} }} } \right]e^{{ - \left( {\alpha_{{\text{b}}} + \gamma \beta_{{\text{b}}} } \right)D - \beta_{{\text{b}}} D^{2} }}$$

where *δ* is the maximum number of the lethal lesions (LLs) per cell nucleus induced by NTEs, *α*_b_ and *β*_b_ are the proportionality factors for the NTEs to *D* [Gy] and *D*^2^ [Gy^2^], respectively. These parameters represent the probabilities of target activation for releasing the cell-killing signals from radiation hit cells.

The IMK model for NTEs (Eq. (4)) was further developed so as to reproduce the experimental synergetic effects between 4-MU treatment and X-ray irradiation. We assumed that the cell-specific parameter *δ* representing maximum level of intercellular signalling effects depends on the 4-MU concentration because the bystander effects are intrinsically related with the inflammatory responses, such as NF-kB and COX-2^41^. In the same manner as Eq. (1), we describe *δ* as a function of 4-MU concentration in μM as

5 $$\delta = \delta_{\min } + \frac{{\delta_{\max } - \delta_{\min } }}{{1 + (D_{{\text{P}}} /ED_{50} )^{ - r\delta } }}.$$

where *δ*_min_ is the minimal *δ* value at *D*_P_ = 0 μM, *δ*_max_ is the maximum *δ* value, *D*_P_ is the 4-MU concentration in μM, *ED*_50_ is effective dosage leading to median *δ* value, and *r*_δ_ is the hillslope. In addition, we assumed that the *ED*_50_ is the common parameter in Eq. (1).

Using Eqs. (2)–(5), we estimated the cell surviving fraction after irradiation under 4-MU treatment. The set of model parameters (*α*_0_, *β*_0_, (*a* + *c*), *α*_b_, *β*_b_, *δ*_min_, *δ*_max_, *r*_δ_) can be determined by fitting the model to the experimental survival curves after irradiation for various conditions of 4-MU administration.

### Overall cell surviving fraction after irradiation in combination with drug

We thirdly express the overall cell surviving fraction using Eqs. (1)–(5). It can be assumed that the mechanisms inducing cell-killing in pharmacological effects (i.e., growth arrest) is independent of that in radiation responses (i.e., DNA damage responses). Multiplying the cell surviving fraction for pharmacological effects *S*_P_, that for DNA-TEs *S*_T_ and that for NTEs *S*_NT_, the overall cell surviving fraction, *S*, can be given by

6 $$S = S_{{\text{P}}} \times S_{{\text{T}}} \times S_{{{\text{NT}}}} .$$

Using Eqs. (1)–(6), we estimated the surviving fraction for various conditions of irradiation and drug administration to evaluate the curative effects under 4-MU treatment in chemoradiation therapy.

### Determination of the parameters in the IMK model

The set of model parameters in the IMK model (*S*_Pmin_, *ED*_50_, *r*_P_, *α*_0_, *β*_0_, (*a* + *c*), *α*_b_, *β*_b_, *δ*_min_, *δ*_max_, *r*_δ_) was determined by simultaneously fitting the model to the experimental survival data. It should be noted that the microdosimetric quantity, *g*, was obtained from our previous study using the Monte Carlo simulation^36^. In addition, considering that the mean (*a* + *c*) value of cancer cells is ranging from 2.18 to 2.23 in the literature^38^, we adopted a value in this range to (*a* + *c*) as a prior information in the fitting procedure of the model to the experimental data. The experimental survival data used for the fitting are: (i) cell surviving fraction as a function of 4-MU concentration, (ii) dose–response curve on cell survival by radiation for three 4-MU concentration cases of 0 μM, 80 μM and 100 μM, and (iii) surviving fraction after constant 4 Gy irradiation under various 4-MU concentrations. Note that the number of experimental datasets for the fitting is 37. The model includes 11 cell-specific parameters as free parameters, and we determined the parameter values by means of maximum likelihood method with a Monte Carlo technique. After determining the model parameters, we estimated the surviving fraction after 4-MU treatment and/or X-ray irradiation.

### Fit quality

To check the performance of the present IMK model (Eqs. (1)–(6)), we calculated the determination coefficient of *R*^2^ expressed as

7 $$R^{2} = 1 - \frac{{\sum_{i = 1}^{n} (\exp_{i } - {\text{ cal}}_{i } )^{2} / (n - m - 1)}}{{\sum_{i = 1}^{n} (\exp_{i } - < \exp > )^{2} / (n - 1)}},$$

where *n* is the number of the experimental data, exp_*i*_ is measured cell survival, cal_*i*_ is cell survival calculated by the model, < exp > is the mean of measured cell survival, *m* is the number of model parameter.

## Results and discussion

### Measured cell survival under 4-MU treatment

**Figure 1:**
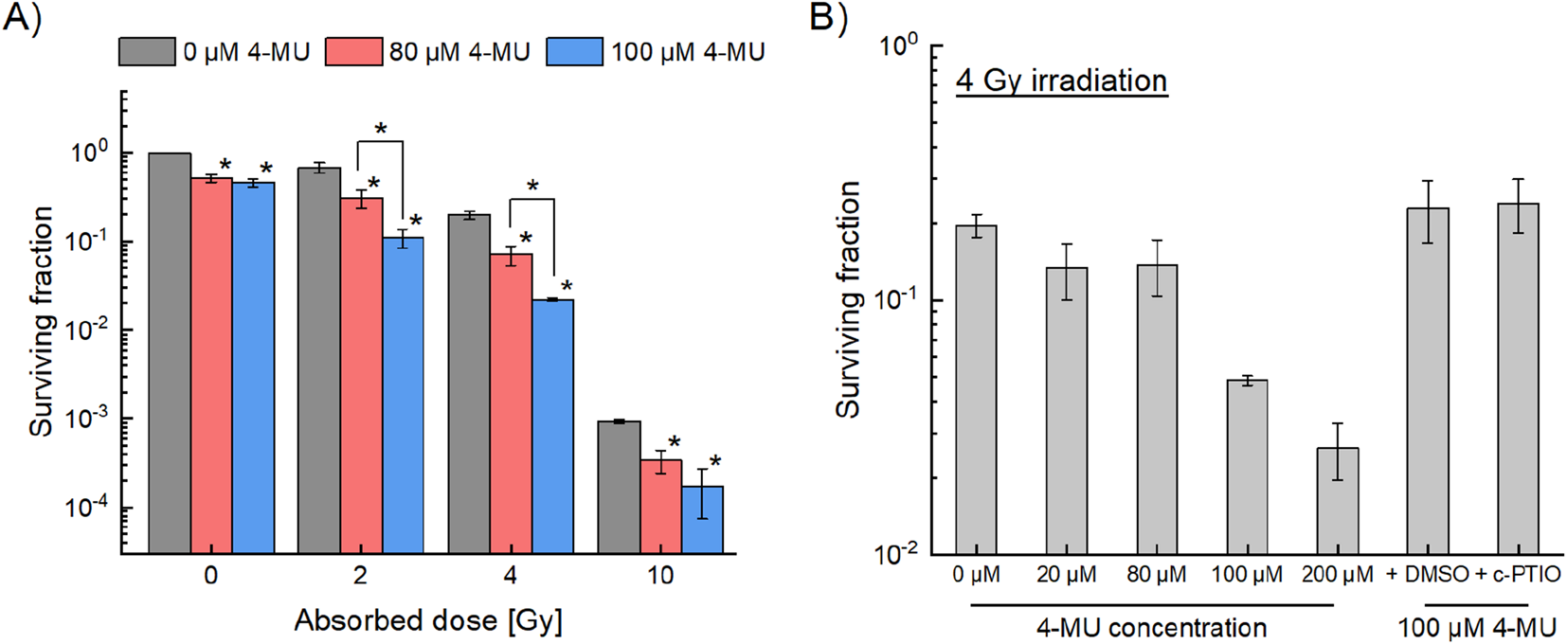
Measured cell survival fraction of HT1080 treated with 4-MU and X-ray irradiation. (**A**) The logarithmic surviving fraction of HT1080 cells irradiated with 0, 2, 4 and 10 Gy under the 4-MU concentrations of 0, 80 and 100 μM, and (**B**) the survival of 4 Gy irradiation with additional 4-MU concentrations of 20 and 200 μM, and 100 μM 4-MU with the ROS inhibitor (1% DMSO) or the NO inhibitor (40 μM c-PTIO). Note that (*) on the bar graph represents p < 0.05 compared to the data under the 0 μM 4-MU, and bracketed asterisks represent significant differences of p < 0.05 between the two groups.

To investigate the impact of 4-MU treatment on radiosensitization, we first measured the surviving fraction of HT1080 cells by means of clonogenic survival assay. Figure 1A shows the relation between absorbed dose and surviving fraction of HT1080 cells under the administrations of 0, 80, and 100 μM 4-MU. The surviving fraction in the presence of the 80 μM 4-MU is significantly lower than that in the 0 μM 4-MU. Intriguingly, the decrease of the cell survival was more remarkable in the 100 μM 4-MU compared to the 80 μM 4-MU at intermediate dose range of 2–4 Gy, whilst there was no significant difference between the 80 μM 4-MU and the 100 μM 4-MU at the high dose of 10 Gy (Fig. 1A). These results suggest that the radiosensitivity administered by 4-MU can be enhanced in the intermediate dose range around 2–4 Gy.

The radiosensitizing effects of 4-MU are intrinsically related with the suppression of antioxidant activity through the anti-inflammatory effects^11^. In this regard, we next measured the cell survival after 4 Gy irradiation for various 4-MU concentrations. As shown in Fig. 1B, the cell-killing effects under the 100 μM and 200 μM 4-MU treatments were significantly enhanced, which suggests that the synergetic effects can be obtained for the 4-MU treatment with the concentration of > 100 μM. In addition, suspecting the involvement of intercellular communication under 4-MU treatment, we also measured the cell survivals in the presence of 1% DMSO as ROS inhibitor and 40 μM c-PTIO as nitric oxide (NO) inhibitor (Fig. 1B). As expected, the synergic effects between the 100 μM 4-MU and 4 Gy irradiation were diminished by these inhibitors.

Since both ROS and NO play important roles in NTEs transmitters^42,43^ as shown in Fig. 1, we speculated that the 4-MU treatment modulates the radiosensitivity by activating NTEs (not by a simple addition of killing effects to 4-MU and X-ray treatments). In addition, as shown in Fig. 1B, there was no significant difference between the results of 1% DMSO and 40 μM c-PTIO, suggesting that NO as a mediator of NTEs is the key factor in the radiosensitizing mechanism of 4-MU. As reported previously, inducible NO synthase (iNOS) produces NO in irradiated cells, inducing not only DNA damage induction through the TGF-β1 pathway^44^ but also radioresistance^45^. Amongst these biological effects induced by intercellular signals, the intermediate dose such as 2–4 Gy exhibits remarkable cell-killing effects by NTEs^46,47^. This is most likely because it exceeds the capacity of the receiving cell own anti-oxidant and DNA repair. Exosome-like vesicles including mitochondrial DNA play an important role in cell-killing effects by NTEs^41,47^, while including microRNA-155 diminished via suppression of NOS^48^. Based on these reports, these exosomes may be a key molecular target for intercellular signalling under 4-MU administration. Meanwhile, the experimental results in Fig. 1 suggest that concentration over 100 mM can sufficiently reduce the tolerance of signal-receiving cells in addition to the toxicity of 4-MU itself. The experimental results revealed that the main cause inducing the synergetic cell-killing effects might be the NTEs by 4-MU, whilst the mechanisms for radioresistance remain to be fully clarified. The further in vitro experiments focusing on iNOS are needed in the future study.

### Theoretical analysis of the cell survival under 4-MU treatment by the IMK model

To theoretically reproduce the experimental cell survival under 4-MU treatment (Fig. 1), we used the modified IMK model considering pharmacological effects (Eq. (1)), DNA-TEs (Eqs. (2) and (3)) and intercellular communication (Eqs. (3)–(5))^32,37^. The parameters in the present model were summarized in Table 1. Using the parameters and Eqs. (1)–(6), we estimated the cell survivals for various conditions of 4-MU treatments and X-ray irradiation.

**Table 1:** Model parameters of the IMK model.

Model parameters		Unit	Values	
			Mean	s.d
Pharmacological effects	*S*_min_	–	0.0003	0.0002
	*S*_max_	–	1.000	–
	*ED*_50_	μM	92.07	1.797
	*r*_d_	–	1.637	0.012
Microdosimetric quantity	*y*_*D*_	keV/mm	4.683	0.050
	*g*	Gy	0.954	0.011
DNA-targeted effects	*α*_0_	Gy^−1^	0.053	0.028
	*β*_0_	Gy^−2^	0.069	0.004
	*a* + *c*	h^−1^	2.215	0.007
Intercellular communication	*α*_b_	Gy^−1^	0.010	0.006
	*β*_b_	Gy^−2^	0.032	0.018
	*δ*_min_	–	0.760	0.016
	*δ*_max_	–	8.175	< 0.001
	*r*_δ_	–	8.816	0.011

**Figure 2:**
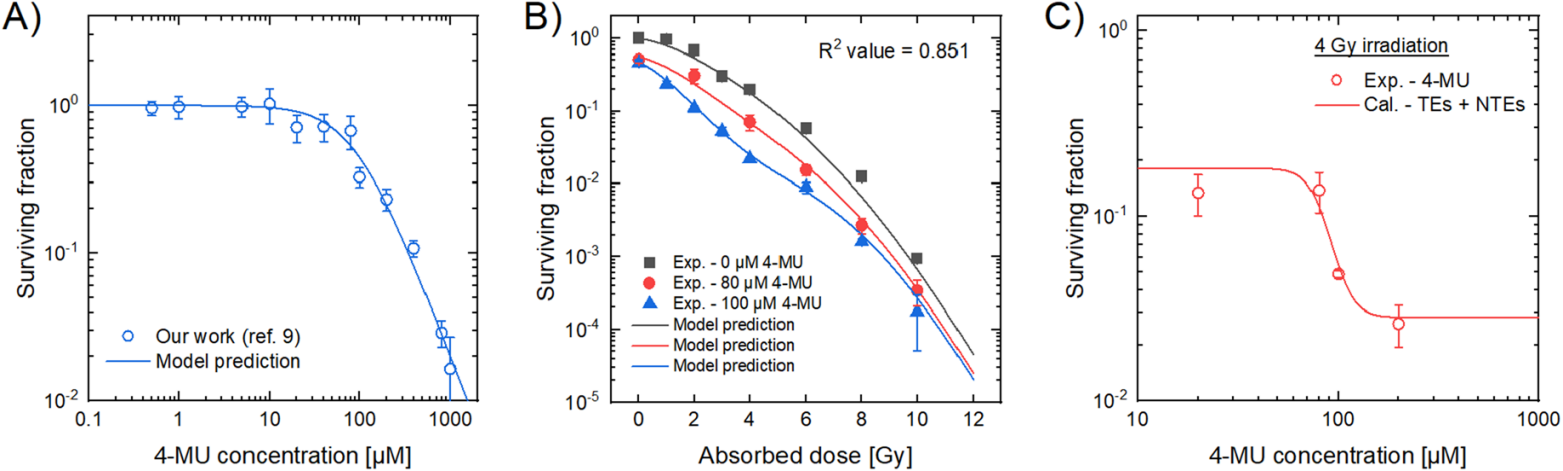
The cell surviving fraction estimated by the model. (**A**) The dose–response curve for 4-MU concentration of HT1080 cells. Blue solid line represents the survival fraction estimated by the log-logistic model (Eq. (1)), blue circle represents the experimental surviving fraction^9^. (**B**) The surviving fraction as a function of absorbed dose. R^2^ value was calculated using Eq. (7). Charcoal line, red line and blue line represent the model prediction for the 0, 80 and 100 μM 4-MU cases, respectively. (**C**) The cell survival curve as a function of 4-MU concentration with a constant 4 Gy irradiation. Red line represents the IMK model prediction and red circles denote experimental data.

These model parameters determined by fitting Eqs. (1)–(6) to the experimental survival (Fig. 2) by means of maximum likelihood method. The values were presented mean ± fitting error (s.d.).

To check the performance of pharmacological part in the IMK model, we compared the model estimation with the experimental result for the relation between 4-MU concentration and cell survival^9^ (Fig. 2A). As shown in Fig. 2A, the model estimation indicates that the effective dosage inducing 50% cell death, *ED*_50_, was 92.07 ± 1.80 μM. Figure 2B and C show the comparison of cell surviving fraction between the model estimation and the experimental data, where Fig. 2B is the dose–response curve by irradiation for 0, 80 and 100 μM 4-MU cases, and Fig. 2C is the survival curve as a function of 4-MU at the constant 4 Gy irradiation. From these comparisons, the modified IMK model agreed well with the experimental data reported in the previous study^9^ and in the present experimental data. From a trend of the curve in Fig. 2C, the parameter *δ* should be a function of the 4-MU concentration. The *δ* value represents the NTEs-induced lethal DNA damage which can lead to cell death, supporting the synergetic effects (from 4-MU treatment and irradiation) predominantly attributed to intercellular signaling^32^.

The dose–response curves on cell survival of HT1080 cells administered with 0, 80 and 100 μM 4-MU were also reproduced by using the present IMK model (Eqs. (1)–(6)) and the model parameters (Table 1), as shown in Fig. 2B. The linear-quadratic (LQ) model is a simplified biological model and a general estimation approach in radiotherapy^49^. Thus, we also compared the fit quality of the LQ model to that of the present IMK model. As a result, we confirmed that the present IMK model considering the NTEs was in better agreement with experimental data than the LQ model (see Figure S1 and Table S1 in supplementary data). Therefore, the consideration of the NTEs in cell-killing model is of importance to predict the curative effects under 4-MU treatment.

**Figure 3:**
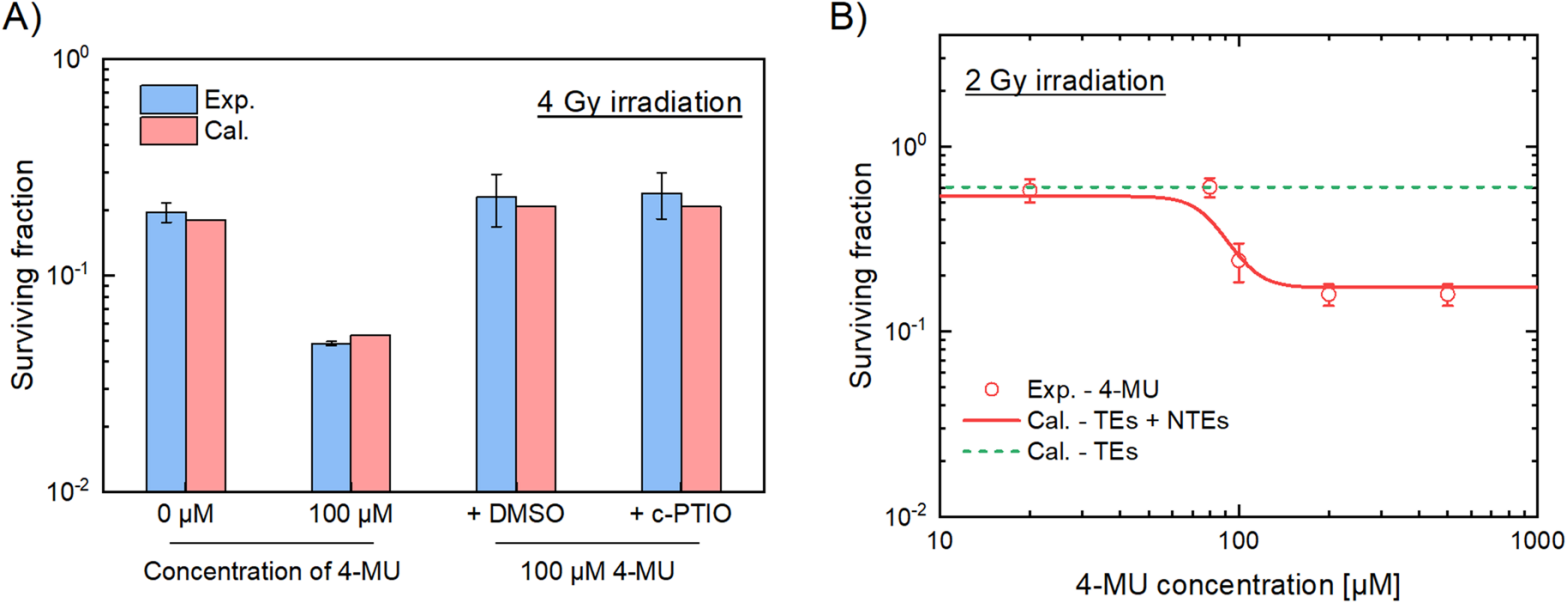
Additional verification of the IMK model in comparison with the experimental data. (**A**) The surviving fraction of HT1080 cells was treated with the 100 μM 4-MU and the ROS inhibitor (1% DMSO) or NO inhibitor (40 μM c-PTIO). We estimated surviving fraction based on the IMK model and the model parameters listed in Table 1. (**B**) The surviving fraction as a function of the 4-MU concentration with a constant 2 Gy irradiation. Red solid line represents the surviving fraction estimated by the IMK model, red circle the experimental cell survival treated with the 100 μM 4-MU, and green dot line the cell survival estimated by the IMK model considering only DNA-TEs.

To further show the validation of the present IMK model (Eqs. (1)–(6)), here we add the comparison between the model predictions and the corresponding experimental data in the presence of NTEs inhibitors. As shown in Fig. 3A, for the groups treated with 4-MU and 4 Gy X-ray irradiation, the surviving fraction of the cells treated with 1% DMSO or 40 μM c-PTIO agreed well with the model prediction considering only TEs with *δ* = 0. In addition, Fig. 3B shows the survival fraction as a function of the 4-MU concentration at a constant 2 Gy irradiation. These comparisons clearly exhibit that the IMK model in this study fairly reproduces the experimental results.

### Oxidative stress level under 4-MU administration

**Figure 4:**
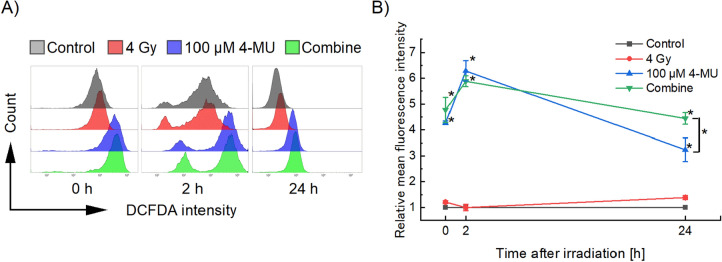
Flow cytometric patterns for the intracellular oxidative stress level. (**A**) Representative histograms and (**B**) the relative mean fluorescence intensity of the HT1080 cells. The mean fluorescence intensity of the 4 Gy irradiation alone group, 100 μM 4-MU treatment alone group, and combine group are standardized by the mean fluorescence intensity of the control group at each time. Note that asterisk (*) on the plot represents p < 0.05 compared to the 4 Gy irradiation alone group, and bracketed asterisk represents significant differences of p < 0.05 between the two groups.

To further evaluate the NTE-related radiosensitizing effects under 4-MU administration, the intracellular overall oxidative stress levels (i.e., ROS and NO production levels) were measured by using flowcytometry after 4 Gy X-ray irradiation in the presence of 100 μM 4-MU (Fig. 4 and Table 2). At 0 h after the treatment, the oxidative stress levels increased 1.2-fold for 4 Gy irradiation alone group, 4.2-fold for 4-MU alone group, and 4.8-fold for combined group, compared to the control group. At 2 h after the treatment, the 4 Gy irradiation alone group decreased to the same level as the control group, whereas the level drastically increased under 4-MU administration. Especially, at 24 h after the treatment, 4-MU administration alone group and combine group decreased whilst the 4 Gy irradiation alone group increased to 1.4-fold. As shown in the previous studies^50,51^, the radiation-induced oxidative stress level occurs immediately after irradiation, then the antioxidant activity such as superoxide dismutase and the expression of Nrf2 (and its downstream pathway) increased. Regarding this, the administration of 4-MU conduced to a contrast with the results of 4 Gy irradiation at the peak of the oxidative stress level, suggesting that 4-MU inhibits antioxidant activity. The later peak after irradiation is interpreted as a sign that the secondarily generated ROS involve mitochondria and enzymatic activity (i.e., NADPH oxidase), but it remains controversial^52–54^. The combine group had a significantly higher oxidative stress level at 24 h than the 4-MU administration alone group, indicating that the secondary-induced ROS by the 4-MU treatment enhance the radiation effects. Shao et al. have reported that the NO level produced by iNOS in irradiated cells elevated until 24 h after irradiation^46^, and DNA damage can be induced by reactive nitrogen species like a peroxynitrite which is the reaction product by NO and superoxide^55^. Based on these previous reports, the secondary ROS measured in this study might be involved in NO, leading to the synergetic cell-killing effects under the 4-MU administration and ionizing irradiation. However, further in vitro studies for antioxidant activity and NO production by 4-MU are necessary to confirm this aspect.

**Table 2:** Raw data of relative mean fluorescence intensity measured by flowcytometry.

Sample	Relative mean fluorescence intensity					
	0 h		2 h		24 h	
	Mean	s.d	Mean	s.d	Mean	s.d
Control	1.00	0.00	1.00	0.00	1.00	0.00
4 Gy	1.21	0.05	1.00	0.10	1.39	0.07
100 μM 4-MU	4.27	0.06	6.28	0.32	3.24	0.37
Combine	4.77	0.40	5.88	0.17	4.23	0.18

DCFDA fluorescence intensity 0, 2, and 24 h after treatment is shown.

### The cell survival fraction for two clinical regimens

The fibrosarcoma cells (HT1080) showed remarkable cell-killing effects especially in the intermediate dose range of around 4 Gy (Figs. 2, 3 and 4) at 4-MU concentration, which is not toxic to normal tissues^9^. To see the possibility of 4-MU in clinical application, we finally calculated the cell surviving fraction hypothetically combined treatment with the 80 μM 4-MU or the 100 μM 4-MU by the IMK model (Eqs. (1)–(6) and Table 1) for two clinical regimens, i.e., 2 Gy/fraction as conventional scheme and 10 Gy/fraction as stereotactic radiotherapy scheme. Both of fractionated regimens were compared to each other at the dose to achieve the 10^–5^ survival level (*S*_c_ = 10^–5^ as an example) that is required for local control of the cancer in radiotherapy^56–58^. The cell survival *S*_T_ and *S*_NT_ in the case of fractionated irradiation were expressed as

8 $$- \ln S_{T} = \mathop \sum \limits_{i = 1}^{n} \left[ {\left( {\alpha_{0} + \gamma \beta_{0} } \right)d + F\beta_{0} d^{2} } \right]$$

9 $$- \ln S_{{{\text{NT}}}} = \mathop \sum \limits_{i = 1}^{n} \left[ {\delta \left[ {1 - e^{{ - \left( {\alpha_{b} + \gamma \beta_{b} } \right)d - \beta_{b} d^{2} }} } \right]e^{{ - \left( {\alpha_{b} + \gamma \beta_{b} } \right)d - \beta_{b} d^{2} }} } \right]$$

where *n* is number of fractions, *d* is the dose per fraction in Gy. Here, we assumed that the lethal lesions by NTEs (or hyper-radiosensitivity) can be accumulated during fractionated irradiations at 24 h interval, resting on the experimental reports^59–61^. Overall cell survival in fractionated irradiation considering the pharmacological effects was given by Eq. (6) with Eqs. (8) and (9). It should be noted that the proliferation between fractionated irradiations is not considered.

**Figure 5:**
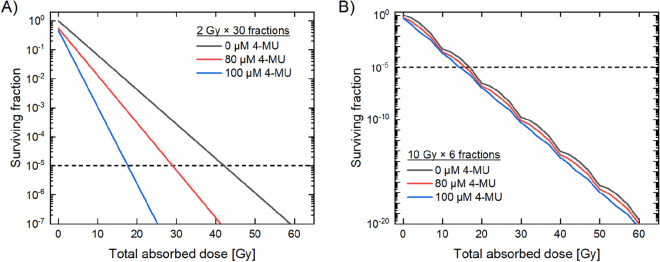
The cell survival during fractionated irradiation under 4-MU administration. Dose–response curve of HT1080 cells for each 4-MU concentration irradiated by (**A**) 2 Gy/fraction scheme or (**B**) 10 Gy/fraction scheme. Charcoal line is the dose–response curve of the non-treated cells, red line is the curve of the 80 μM 4-MU treated cells, and blue line is the curve of the 100 μM 4-MU treated cells. The horizontal black dotted line represents the surviving fraction *S*_c_ = 10^–5^.

From the estimation results for the case of 2 Gy per fraction (Fig. 5A), the total doses 39.92 Gy, 34.91 Gy and 16.38 Gy are required to achieve *S*_c_ = 10^–5^ for the non-treated cells (control), those treated with the 80 μM 4-MU and those treated with the 100 μM 4-MU, respectively. Noteworthy, in the presence of the 80 μM 4-MU and 100 μM 4-MU, the model exhibits that *S*_c_ can be achieved with much less than the dose compared with control for the case of 2 Gy. In contrast, for the case of 10 Gy per fraction (Fig. 5B), there is a slight difference in the *S*_c_ dose amongst the three 4-MU concentrations. The results suggest that the NTE is influential at 2 Gy/fraction in the presence of 4-MU as previously shown in Figs. 1, 2. The 4-MU administration is expected to greatly enhance the curative effects of fibrosarcoma under the regimen of 2 Gy/fraction in conventional radiotherapy. However, because the tumour repopulation^62^ was not considered in the model, further verification tests of the model are necessary by taking account of the corresponding experimental data and also a variety of fractionation numbers.

## Conclusion

This work shows the involvement of intercellular communication in radiosensitizing effects under the 4-MU treatment, from the viewpoints of both cell experiments and model analyses. The results showed that the enhancement of cell killing by 4-MU treatment is the greatest in the intermediate dose range around 4 Gy, which is attributable to intercellular communication. The impact associated with NTEs (mainly NO) was also supported by oxidative stress detection assay. The pharmacological effects and radiation effects were successfully described by the integrated theoretical cell-killing model, which would be beneficial as an estimation tool for chemoradiotherapy with 4-MU. However, the molecular mechanisms that 4-MU-induced secondary ROS leads to the synergistic effects is not yet be fully elucidated. Further study that can handle clinical situations is needed for translating from in vitro to in vivo or in situ.

Whilst further investigations of underlying mechanisms on radioresistance still remain, the present in vitro investigation and modelling reveal that the chemoradiotherapy with 4-MU treatment would be promising to provide more efficient tumour control than the conventional X-ray therapy.

## Supplementary Information

Supplementary Information.
