# Supplementary material for: 4-Methylumbelliferone administration enhances radiosensitivity of human fibrosarcoma by intercellular communication
Source: Sci Rep. 2021 Apr 15;11:8258. doi: 10.1038/s41598-021-87850-3 (PMC8050271; doi:10.1038/s41598-021-87850-3)
Supplement: Supplementary file 1 — Supplementary Information. [file 41598_2021_87850_MOESM1_ESM.docx]

# **Supplementary data:**

# **4-Methylumbelliferone administration enhances radiosensitivity of human fibrosarcoma by intercellular communication**

Ryo Saga, Yusuke Matsuya, Rei Takahashi, Kazuki Hasegawa, Hiroyuki Date, Yoichiro Hosokawa





**Figure S1. Comparison between the LQ model and the IMK model.** The dose-response curve of HT1080 cells treated with A) 0 μM 4-MU, B) 80 μM 4-MU, and C) 100 μM 4-MU. Blue dotted line and blue solid line represent the surviving fraction calculated by the LQ model and the IMK model, respectively. The blue circle plot is the measured cell survival. It should be noted that the LQ model is depicted after fitting the model to the experimental data for each 4-MU concentration, whilst the curve by the IMK model is obtained from the main paper.

| **4-MU concentration** | **Type of cell-killing model** | **Statistical index** |
| --- | --- | --- |
|  |  | ***χ*^2^ value** |
| 0 μM | Linear-Quadratic (LQ) model | 3.57 × 10^2^ |
|  | Integrated MK (IMK) model | 1.91 × 10^2^ |
| 80 μM | Linear-Quadratic (LQ) model | 2.99 × 10^0^ |
|  | Integrated MK (IMK) model | 8.41 × 10^-1^ |
| 100 μM | Linear-Quadratic (LQ) model | 4.47 × 10^1^ |
|  | Integrated MK (IMK) model | 8.75 × 10^0^ |
| Total | Linear-Quadratic (LQ) model | 4.04 × 10^2^ |
|  | Integrated MK (IMK) model | 2.00 × 10^2^ |

**Table S1. Fit qualities of the LQ model and the IMK model to the experimental data.** The *χ*^2^ value was used as a statistical index for evaluating the model performance. Note that the model predictions and the experimental data were depicted in Fig. S1.
